# Single-nucleotide polymorphism, linkage disequilibrium and geographic structure in the malaria parasite *Plasmodium vivax*: prospects for genome-wide association studies

**DOI:** 10.1186/1471-2156-11-65

**Published:** 2010-07-13

**Authors:** Pamela Orjuela-Sánchez, Nadira D Karunaweera, Mônica da Silva-Nunes, Natal S da Silva, Kézia KG Scopel, Raquel M Gonçalves, Chanaki Amaratunga, Juliana M Sá, Duong Socheat, Rick M Fairhust, Sharmini Gunawardena, Thuraisamy Thavakodirasah, Gawrie LN Galapaththy, Rabindra Abeysinghe, Fumihiko Kawamoto, Dyann F Wirth, Marcelo U Ferreira

**Affiliations:** 1 Department of Parasitology, Institute of Biomedical Sciences, University of São Paulo, 05508-900 São Paulo, São Paulo, Brazil; 2 Department of Parasitology, Faculty of Medicine, University of Colombo, Kinsey Road, Colombo 8, Sri Lanka; 3 Center of Health Sciences, Federal University of Acre, 69915-900 Rio Branco, Acre, Brazil; 4 Institute of Biological Sciences, Federal University of Juiz de Fora, 36036-330 Juiz de Fora, Minas Gerais, Brazil; 5 Laboratory of Malaria and Vector Research, National Institute of Allergy and Infectious Diseases, National Institutes of Health, Rockville, MD 20852, USA; 6 National Malaria Center, Phnom Penh 1, Cambodia; 7 Anti-Malaria Campaign, Ministry of Health, Colombo 10, Sri Lanka; 8 Department of Social and Environmental Medicine, Institute of Scientific Research, Oita University, Oita 879-5593 Japan; 9 Department of Immunology and Infectious Diseases, Harvard School of Public Health, Boston, MA 02115, USA

## Abstract

**Background:**

The ideal malaria parasite populations for initial mapping of genomic regions contributing to phenotypes such as drug resistance and virulence, through genome-wide association studies, are those with high genetic diversity, allowing for numerous informative markers, and rare meiotic recombination, allowing for strong linkage disequilibrium (LD) between markers and phenotype-determining loci. However, levels of genetic diversity and LD in field populations of the major human malaria parasite *P. vivax *remain little characterized.

**Results:**

We examined single-nucleotide polymorphisms (SNPs) and LD patterns across a 100-kb chromosome segment of *P. vivax *in 238 field isolates from areas of low to moderate malaria endemicity in South America and Asia, where LD tends to be more extensive than in holoendemic populations, and in two monkey-adapted strains (Salvador-I, from El Salvador, and Belem, from Brazil). We found varying levels of SNP diversity and LD across populations, with the highest diversity and strongest LD in the area of lowest malaria transmission. We found several clusters of contiguous markers with rare meiotic recombination and characterized a relatively conserved haplotype structure among populations, suggesting the existence of recombination hotspots in the genome region analyzed. Both silent and nonsynonymous SNPs revealed substantial between-population differentiation, which accounted for ~40% of the overall genetic diversity observed. Although parasites clustered according to their continental origin, we found evidence for substructure within the Brazilian population of *P. vivax*. We also explored between-population differentiation patterns revealed by loci putatively affected by natural selection and found marked geographic variation in frequencies of nucleotide substitutions at the *pvmdr-1 *locus, putatively associated with drug resistance.

**Conclusion:**

These findings support the feasibility of genome-wide association studies in carefully selected populations of *P. vivax*, using relatively low densities of markers, but underscore the risk of false positives caused by population structure at both local and regional levels.

See commentary: http://www.biomedcentral.com/1741-7007/8/90

## Background

*Plasmodium vivax*, the most widespread of the four human malaria parasites, causes 132 to 391 million episodes of disease each year [1], with 2.6 billion people at risk of infection worldwide [2]. Outside of Africa, *P. vivax *is the main cause of malaria morbidity, with enormous public health burden. The recent emergence of drug-resistant strains and severe (sometimes fatal) disease challenges the traditional view of vivax malaria as a benign infection and calls for new strategies to examine molecular mechanisms underlying drug resistance and increased virulence [3].

Linkage analysis of laboratory crosses and population-based genome-wide association studies are powerful approaches to map genetic loci contributing to drug resistance and virulence in malaria parasites [4] . Experimental crosses of *P. vivax *are currently limited by the lack of practical methods for its long-term propagation and cloning in vitro [5], which are required to characterize phenotypes in the progeny [4]. The ideal populations for allelic association studies are those with high prevalences of the phenotype of interest and relatively high levels of genetic diversity, allowing for numerous informative markers across the genome. Association studies are usually most cost-effective when low meiotic recombination rates are present in these populations, allowing for strong linkage between markers and phenotype-determining loci. The phenotype-associated allele is presumed to have emerged on a single or a few haplotype backgrounds. Crossover events during meiosis tend to randomize the initial haplotype(s) and to reduce population-level associations, known as linkage disequilibrium (LD), between flanking markers and candidate phenotype-determining loci. Therefore, to determine the feasibility of association studies we need a detailed picture of the overall diversity and LD landscape across the genome of *P. vivax*. However, patterns of genetic diversity and LD remain little characterized in field populations of this parasite and most nucleotide polymorphism data currently available are for antigen-coding genes [6]. These data may not be informative of genome-wide patterns because of potential biases introduced by natural selection on particular phenotypes [7]. Due to the lack of continuous culture in vitro, only field samples of *P. vivax*, which are heavily contaminated with host's DNA, are available for use in next-generation sequencing projects for large-scale characterization of new genetic markers.

Microsatellite markers have revealed clear geographic differences in levels of genetic diversity and LD in *P. falciparum *isolates sampled from four continents. Diversity was highest and LD lowest in populations from holoendemic Africa, while diversity was lowest and LD highest in populations from hypoendemic South America, with intermediate patterns seen in Southeast Asia. Parasite populations clustered according to their continental origins, with most variation found within locations in highly endemic areas. Nevertheless, substantial divergence was seen between subpopulations in South America [8]. Putatively neutral microsatellite markers, sampled from across the genome [9], have recently been used to characterize field populations of *P. vivax *[10-12], suggesting that a spectrum of population structures also exists for this species. Again, South American parasite populations sampled from nearby sites are highly divergent [10,11]. However, because the microsatellite markers analyzed map to different chromosomes, prior studies could not examine chromosome-level LD and infer recombination rates in *P. vivax*. In addition, microsatellites mutate at very high rates and other neutral genetic markers with different rate and mode of evolution, such as intergenic or synonymous single-nucleotide polymorphisms (SNPs), could offer a different picture [13]. Significant differences detected with rapidly evolving markers, which may remain undetected with more conserved markers, do not necessarily translate into biologically meaningful differences among populations [14].

To explore the potential for future genome-wide association studies, we examined SNP diversity and LD across a 100-kb chromosome segment of *P. vivax*. We sampled parasites from areas of low to moderate endemicity in South America and Asia, where LD tends to be more extensive than in holoendemic populations. We show varying levels of SNP diversity and LD across populations (highest diversity and LD in the area of lowest malaria transmission, Sri Lanka), with substantial genetic differentiation among populations. Frequencies of nucleotide substitutions at *pvmdr-1 *gene, putatively associated with drug resistance, varied markedly across locations. Although these findings support the use of genome-wide association approaches to map genes underlying drug resistance and other traits in *P. vivax*, they underscore the risk of false positives if population structure, at both local and regional levels, is left uncorrected in association studies.

## Results and Discussion

### Chromosome-level SNP diversity

We examined chromosome-level SNP diversity and LD in 238 field isolates of *P. vivax *from areas of low to intermediate levels of malaria endemicity and two monkey-adapted strains, Belém and Salvador-I. The field isolates originated from three sites in the Amazon Basin of Brazil (Granada, Plácido de Castro and Porto Velho) and three sites (Pursat, Cambodia; Bao Loc, Vietnam; Tricomalee, Sri Lanka) across South and Southeast Asia (Additional file 1 Table S1). We assayed 85 SNPs across 100-kb of contiguous DNA sequence on chromosome 8. Of them, 57 (67.1%) segregated in at least one of the study locations. The number of SNPs that segregated in parasites from Brazil, Cambodia, Sri Lanka and Vietnam were 43, 35, 44 and 24, respectively. Only 13 (15.3%) segregated in all six locations; 8 segregated in Brazil alone and 14 segregated in Asia alone.

We measured SNP π, the average proportion of pairwise differences at assayed SNP loci [15], to compare diversity across study sites; monkey-adapted strains were not considered in this analysis. We obtained the following SNP π values (standard errors in parentheses): Granada = 0.1264 (0.0006); Plácido de Castro = 0.1298 (0.0026); Porto Velho = 0.1589 (0.0110); Brazil (three sites combined) = 0.1364 (0.0005); Cambodia = 0.1163 (0.0012); Vietnam = 0.0842 (0.0034); Sri Lanka = 0.15476 (0.0063); Asia (three sites combined) = 0.1401 (0.0010). The overall SNP π value for Brazilian populations of *P. vivax *is identical to the estimate obtained by Neafsey and colleagues for 11 *P. falciparum *isolates from this country that were assayed for 1638 SNPs across the whole genome [15].

**Figure 1:**
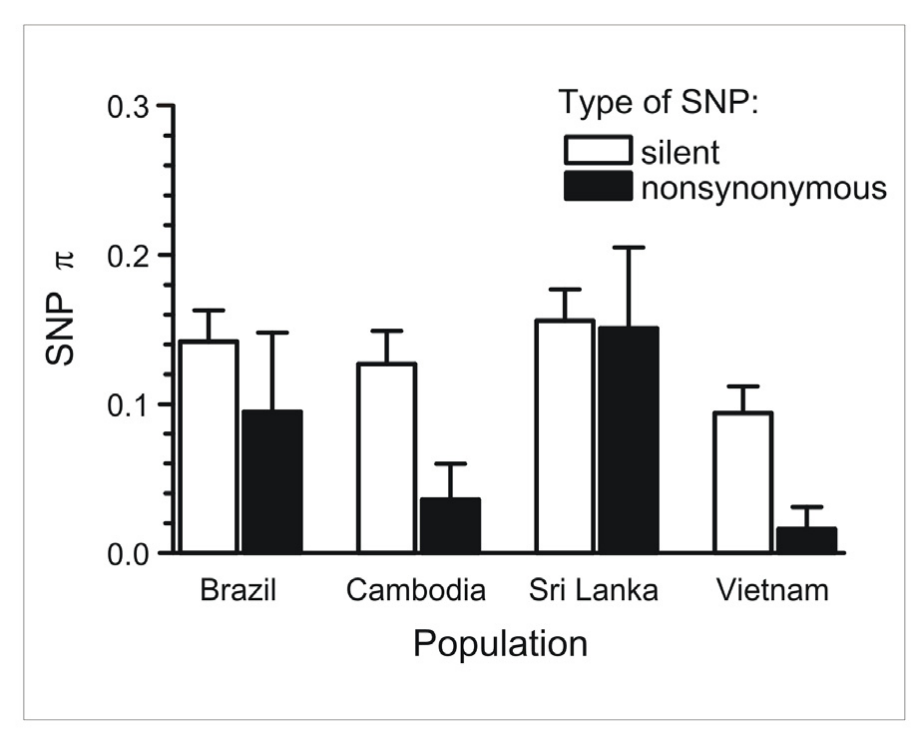
**Diversity at silent and nonsynonymous SNPs (SNP π) across chromosome 8 by *P. vivax *population**. Error bars indicate standard errors of the means, derived from bootstrapping. Significantly lower nonsynonymous SNP diversity was found in all populations but Sri Lanka (*P *< 0.001 by Wilcoxon test).

The observed ranking of country-specific numbers of segregating sites and SNP π values (Sri Lanka > Brazil > Cambodia > Vietnam) does not match the ranking of malaria endemicity at the time of sample collection (Vietnam > Cambodia > Brazil > Sri Lanka). The trend towards a negative correlation between levels of genetic diversity and malaria transmission contrasts with the pattern observed for *P. falciparum*, for which highest diversity is seen in high-transmission settings [8,15]. This contrast is surprising and may reflect differences in the demographic history and biology of these major human parasites. In all countries but Sri Lanka, SNP π values were significantly higher for 75 silent (synonymous or noncoding) SNPs than for 10 nonsynonymous SNPs (Figure 1), consistent with nonsynonymous SNPs being often subject to purifying selection in *P. vivax *populations, as previously suggested for *P. falciparum *[15].

Parasites were systematically sampled over 30 months (April 2004 through October 2006) in one of the study sites in Brazil, Granada [11,16]. We hypothesized that local parasite populations gradually diversify over time as a result of migration, genetic drift, mutation and recombination. Accordingly, the proportion of pairwise SNP differences correlated positively with the temporal distance between dates of collection of pairs of isolates in Granada (*r *= 0.138, *P *< 0.001, Mantel correlation test). The correlation remained significant when only silent SNPs were considered (*r *= 0.146, *P *< 0.001), but not when only nonsynonymous sites were considered (*r *= 0.007, *P *= 0.343). The little variation at nonsynonymous SNP loci over time underscores the potential biases introduced by natural selection in studies of malaria parasite diversity. Whether or not similar temporal patterns of genetic divergence occur in other endemic areas remains to be investigated.

### Linkage disequilibrium and haplotype blocks

**Figure 2:**
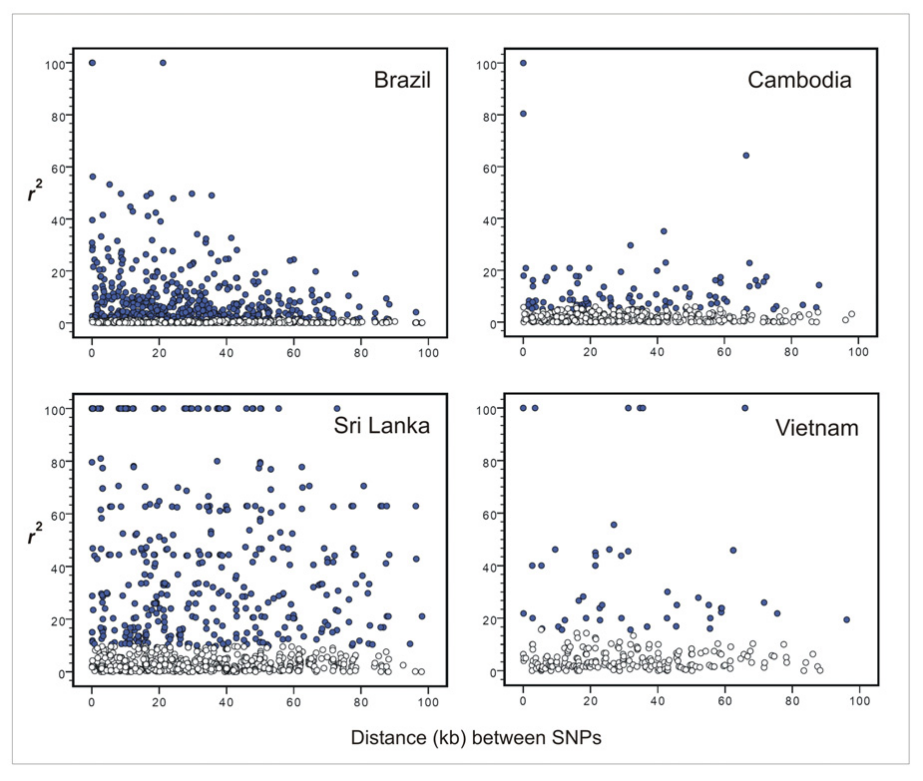
**Variation in LD in relation to physical distance between SNP loci along chromosome 8 of *P. vivax***. Data are shown for Brazil (Granada, Plácido de Castro and Porto Velho combined), Cambodia, Sri Lanka, and Vietnam. LD was measured with the *r*^2 ^statistic (values on y-axis are multiplied by 100); closed (blue) circles denote significant LD (χ^2 ^statistic, *P *< 0.05), while open circles denote nonsignificant LD. A significant negative correlation between *r*^2 ^values and physical distance between SNPs (in kb) was found only for Brazil (Pearson correlation coefficient *r *= -0.195, *P *< 0.0001).

**Figure 3:**
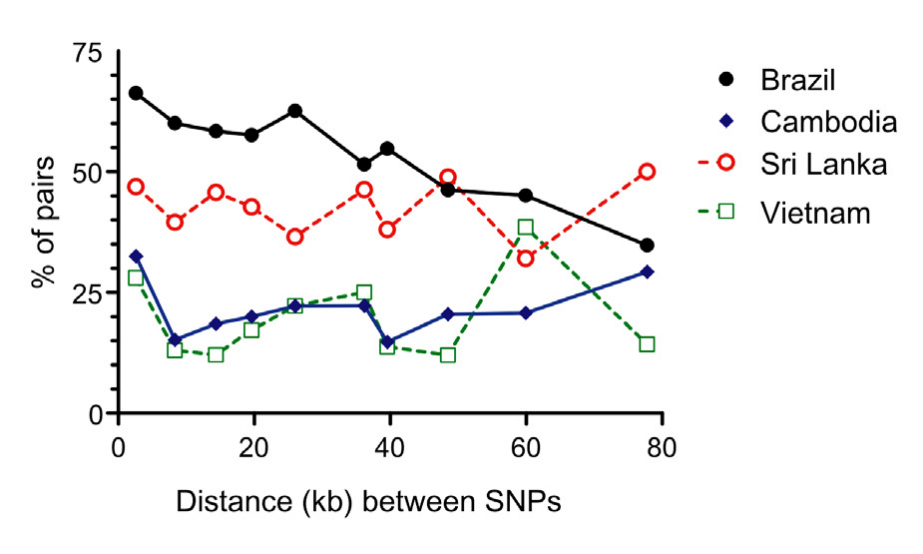
**Proportions of informative SNP pairs that display significant LD plotted at various intermarker distances**. Data are shown for Brazil (Granada, Plácido de Castro and Porto Velho combined), Cambodia, Sri Lanka, and Vietnam. Pairwise physical distances between markers (in kb) were divided into deciles; the average distance within each decile is used to plot values. Note that the proportion of pairs with significant LD decreases with intermarker distance only in Brazil.

The extent of LD between pairs of markers across a chromosome is expected to decline at a rate that is proportional to the population recombination rate. In fact, LD (measured with the *r*^2 ^statistic) decayed with increasing physical distance between pairs of segregating sites in the Brazilian population (Figure 2), with similar results when two subpopulations from this country, Granada and Plácido de Castro, were considered separately to remove the putative effect of population substructuring on LD levels (Additional file 2 Figure S1). However, no significant correlation between *r*^2 ^and intermarker distance was found in Cambodia, Sri Lanka, or Vietnam (Figure 2), with significant LD often extending over the entire chromosome segment analyzed. Accordingly, the proportion of pairs of segregating sites with significant LD declined with increasing map distance in the Brazilian population, but not in populations from areas with the highest endemicity, Cambodia and Vietnam (Figure 3). These results suggest that initial genome-wide association mapping, using relatively low densities of marker loci, is feasible in natural *P. vivax *populations from areas of low to moderate malaria endemicity with the features observed in Brazil, where LD can persist over several kb but clearly declines with increasing intermarker distance. Among parasites from the other sites analyzed, recombination rates may be too low to allow for cost-effective association studies, since the persistence of significant LD over long chromosome segments may lead to frequent false-positive associations.

**Figure 4:**
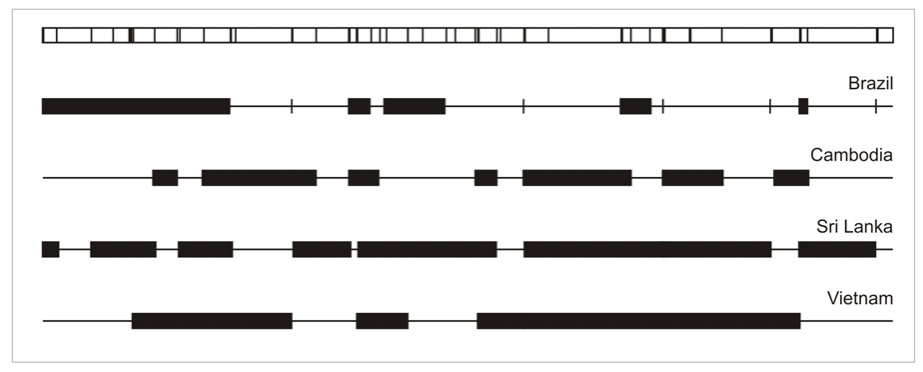
**Haplotype blocks along chromosome 8 of *P. vivax***. On the top, we show the map location of assayed SNPs that segregated in at least one of the four populations (vertical lines, n = 57). Haplotype blocks, denoted by black boxes of different sizes, were defined as clusters of adjacent markers over which little evidence of meiotic recombination was found using the four-gamete rule. Data are shown for Brazil (Granada, Plácido de Castro and Porto Velho combined), Cambodia, Sri Lanka, and Vietnam.

The 100-kb chromosome segment analyzed comprised several clusters of adjacent markers over which little evidence of meiotic recombination was found using the four-gamete rule. These haplotype blocks varied in number and length across the four populations examined (Figure 4). The greatest number of blocks and the smallest average block size (3.6 kb [range, < 100 bp to 21 kb] and 3.4 kb [range, < 100 bp to 9 kb]) were found in Brazil and Cambodia, while the average block sizes for Sri Lanka and Vietnam were 7.4 (range, 1 to 14 kb) and 14.0 kb (range, 5 to 21 kb). Whether the relatively large blocks in these two populations are artefacts resulting from the small sample size remains to be investigated. Haplotype blocks can be further used to define the minimal set of segregating SNPs required to capture most variation in each population, by selecting a single tagging marker within each block (and assaying all SNP loci outside blocks). These minimal sets would comprise 56% (24 of 43) segregating markers in Brazil, 63% (22 of 35) in Cambodia, 43% (19 of 44) in Sri Lanka and 50% (12 of 24) in Vietnam.

We next examined whether the overall haplotype structure was conserved in different populations. To determine to which extent haplotype block boundaries were shared across populations, we calculated the proportions of pairs of markers assigned to the same block (i. e, non-recombining sites) and to different blocks or no block (i. e., recombining sites) in pairwise population comparisons. This analysis was limited to SNPs that were segregating in both populations analyzed (99 to 227 SNP assignments compared). Overall, the vast majority of SNP pairs had a concordant assignment, especially in comparisons involving Brazil and Cambodia (93%), Vietnam and Sri Lanka (91%), and Brazil and Sri Lanka (83%). These data are consistent with a conserved haplotype structure across *P. vivax *populations with varying levels of LD, as previously shown for *P. falciparum *[17]. Lower proportions of concordant assignments were found, however, in comparisons between parasite populations from Vietnam and Cambodia (73%) and Brazil and Vietnam (60%). Most discrepancies were due to marker pairs that were in LD in Sri Lanka and Vietnam but not in Brazil and Cambodia. The vast majority (81-100%) of SNPs with concordant assignment between pairs of populations were silent. We conclude that haplotype block boundaries are shared by parasite populations with different geographic origins, suggesting the existence of conserved recombination hotspots in the genomic region analyzed, with clear implications for future association studies.

### Population structure at local and regional levels

**Figure 5:**
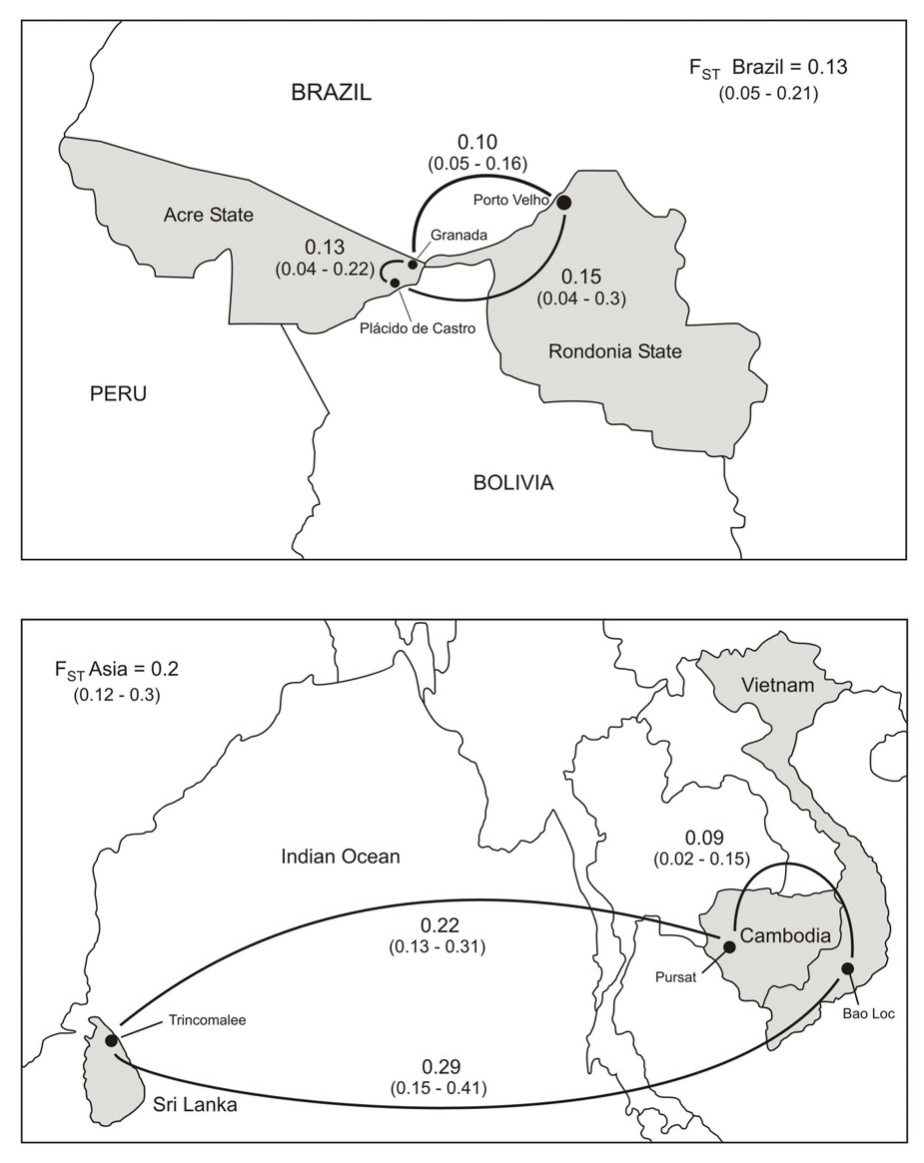
**Population divergence revealed by SNPs along chromosome 8 of *P. vivax***. The map on the top panel shows pairwise estimates of *F*_ST _(with 95% confidence intervals derived from bootstrapping in parentheses) for three populations within Brazil (Granada, Plácido de Castro and Porto Velho), while the lower panel shows pairwise estimates of *F*_ST _for three populations (Pursat, in Cambodia; Trincomalee, in Sri Lanka; and Bao Loc, in Vietnam) across South and Southeast Asia.

Analysis of population differentiation using the θ estimator of *F*_ST _statistics revealed substantial chromosome-level differentiation between Brazilian and Asian samples (*F*_ST _= 0.228, 95% confidence interval [CI] 0.132-0.247). Significant divergence was found in all pairwise comparisons within Brazil and Asia (Figure 5), although relatively low divergence was found between Cambodian and Vietnamese samples, which were collected more than 10 years apart. The overall *F*_ST _value of 0.393 (all locations considered) indicates that a considerable proportion (~40%) of the diversity at assayed SNP loci results from differentiation among geographic populations of *P. vivax*. For all between-population comparisons, except Cambodia versus Sri Lanka, we observed a greater *F*_ST _for silent SNPs relative to nonsynonymous SNPs (Additional file 3 Table S2), suggesting that purifying selection may constrain estimates of population differentiation.

**Figure 6:**
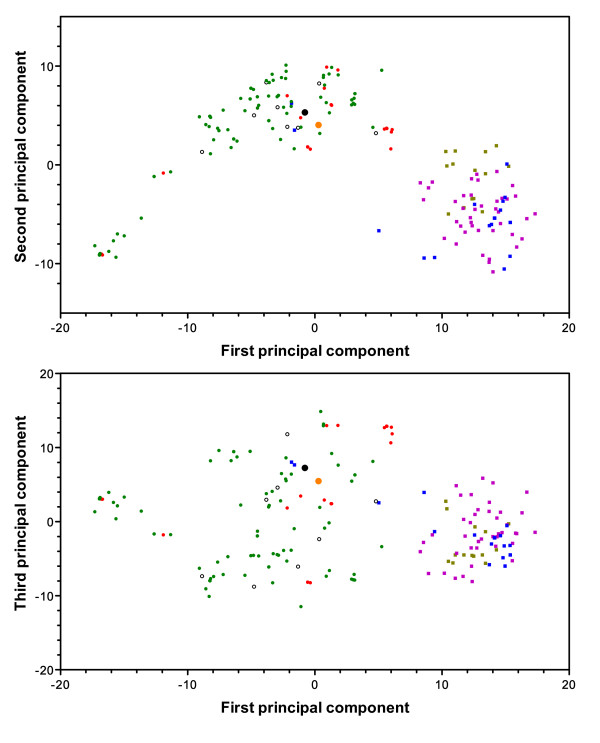
**Principal component analysis (PCA) of population structure of *P*. vivax**. Plots show the three first principal components. Each circle or square represents a parasite isolate or strain and the color is assigned according to the geographic origin of parasites: green circles, Granada; red circles, Plácido de Castro; open circles, Porto Velho (all samples from Brazil are represented with circles); pink squares, Cambodia; blue squares, Sri Lanka; light brown squares, Vietnam (all samples from Asia are represented with squares). The strains Salvador I and Belém are denoted by orange and black circles, respectively. The number of circles or squares plotted in the panels does not correspond to the total number of parasite samples analyzed (Additional file 1 Table S1) because some of them had identical haplotypes and, therefore, had identical PCA coordinates. Upper panel, plot of the first two principal components; Lower panel, plot of the first and third principal components.

Not surprisingly, principal component analysis (PCA) [18] defined two major clusters that reflect the continental origin of samples (Figure 6). The first major cluster, characterized with the first and second principal components (which, together, explain 38% of the variance), comprised all Brazilian samples, the monkey-adapted strains Belém (from Brazil) and Salvador-I (from El Salvador), and two samples from Sri Lanka, whereas the second clusters comprised the remaining Asian samples. By combining the first and third principal components (35% of the variance explained), we observed a greater dispersal of Brazilian samples, a few of which clustered together with Asian samples (Figure 6). We repeated the analysis with a set of 29 SNPs selected to minimize intermarker LD, with quite similar results (Additional file 4 Figure S2), indicating that the observed clustering pattern was not an artifact arising from the interdependence of segregating sites. We also repeated the analysis after excluding the populations with the smallest sample size, Porto Velho and Vietnam, but observed the same clustering pattern, with two isolates from Sri Lanka grouped with those from Granada and Plácido de Castro (data not shown).

As previously shown for *P. falciparum *populations [15,19], PCA provided evidence for substantial substructure within the Brazilian population of *P. vivax*. We thus analyzed separately this population and found that parasites did not cluster according to their collection site (Granada, Plácido de Castro or Porto Velho). In fact, most minor clusters in Brazil comprised samples from at least two of these locations (Additional file 5 Figure S3). We also carried a separate analysis of Asian samples to determine whether further substructuring was apparent. We were unable to differentiate between Cambodian and Vietnamese parasites, but this analysis revealed a large dispersal, with some substructuring, in the Sri Lankan population (Additional file 6 Figure S4). The heterogeneity of parasites from nearby locations in Brazil and from a single outbreak in Sri Lanka indicates that correcting for population structure may be required in future association studies in these endemic sites. Otherwise, there may be a spurious association between a phenotype of interest with varying prevalence among subpopulations (for example, from different locations within the same country) and any candidate genetic marker that display allele frequency differences across these subpopulations.

**Figure 7:**
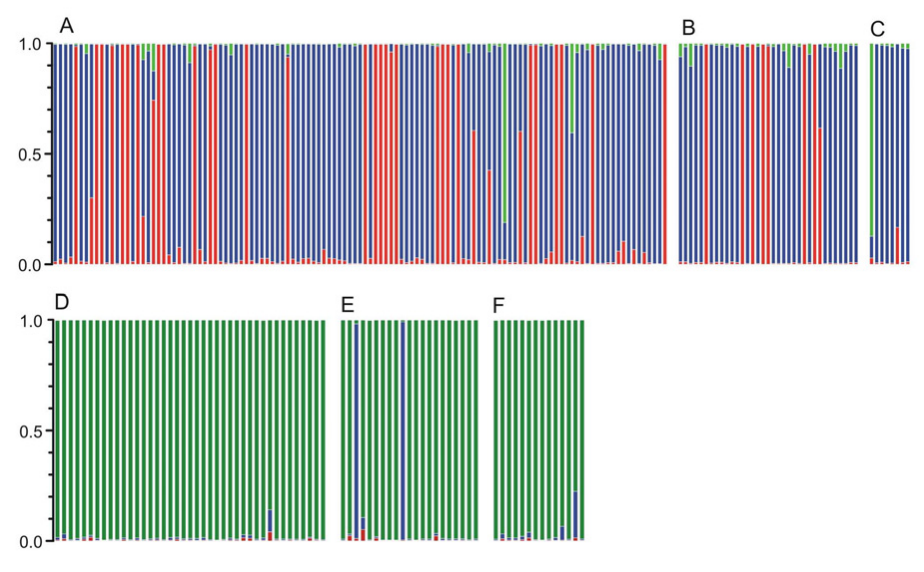
**STRUCTURE analysis of population structure of *P. vivax***. Each bar represents a parasite isolate and bar partitioning represents the proportion of ancestry of each isolate assigned to three populations, represented with different colors (blue, red or green). **A**, Granada; **B**, Plácido de Castro; **C**, Porto Velho (**A**, **B **and **C **are in Brazil); **D**, Cambodia; **E**, Sri Lanka; **F**, Vietnam.

The finding of population structure within Brazil was further supported by the Bayesian clustering procedure implemented by STRUCTURE software [20]. This detected, with strong statistical support (posterior probability > 0.999), three major populations in the whole dataset. Nearly all field isolates from Brazil (in addition to Belém and Salvador-I strains) had predominant ancestry in one of two populations, represented in blue and red in Figure 7. In contrast, nearly all parasites from Asia had their predominant ancestry in a third population, represented in green in Figure 7. Only four isolates failed to cluster according to their continent of origin; exceptions were two isolates from Brazil with a predominant membership in the Asian (green) population and two isolates from Sri Lanka with clear membership in the blue subpopulation from Brazil. Granada and Plácido de Castro, the largest populations in Brazil, comprised parasites with predominant membership in the blue or red populations, while Porto Velho included only parasites with predominant membership in the blue population. A separate STRUCTURE analysis of the Brazilian population confirmed the subdivision into two major populations, without clear further substructuring (data not shown).

### Natural selection and population differentiation

**Figure 8:**
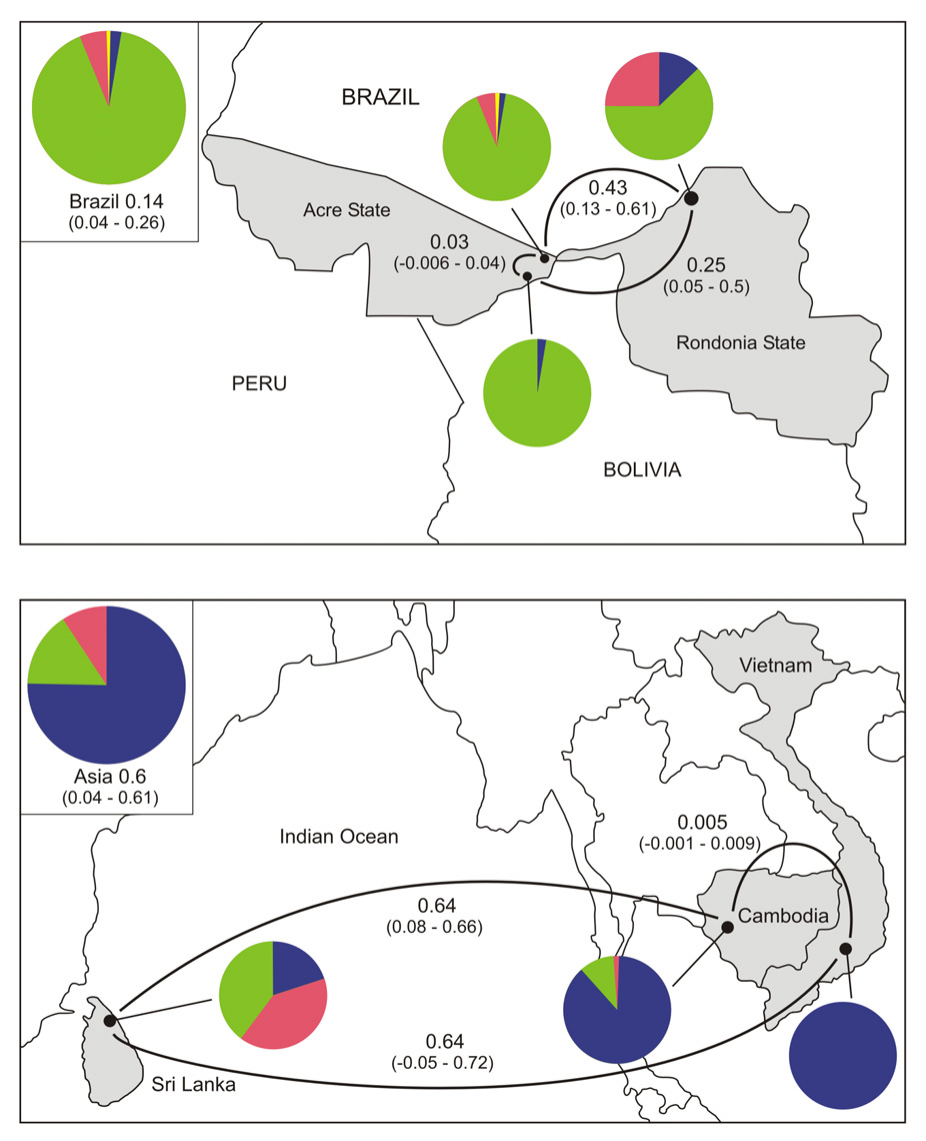
**Population divergence revealed by mutations at the *pvmdr-1 *locus of *P. vivax***. The map on the top panel shows pairwise estimates of *F*_ST _(with 95% confidence intervals derived from bootstrapping in parentheses) for three populations within Brazil, while the lower panel shows pairwise estimates of *F*_ST _for three populations across South and Southeast Asia. Pie charts represent the population-level frequencies of the Y976F and F1076L mutant alleles of *pvmdr-1 *as follows: green, wild type; salmon, F1076L single mutant; yellow, Y976F single mutant; dark blue, Y976F-F1076L double mutant.

We next explored between-population differentiation patterns revealed by loci putatively affected by natural selection. We scored SNPs at two loci, *pvcrt-o *and *pvmdr-1*, encoding digestive-vacuole membrane proteins that can be involved in chloroquine (CQ) resistance in *P. vivax *(Additional file 7 Figure S5). None of the five nonsynonymous SNPs assayed in the *pvcrt-o *gene was found to segregate in any of the parasite populations examined. In contrast, the six SNP sites analyzed within the *pvmdr-1 *gene segregated in most populations. Allele frequencies at *pvmdr-1 *varied markedly between locations, with an overall *F*_ST _of 0.705. We found very little differentiation between neighboring locations within Brazil (Granada versus Plácido de Castro) and Asia (Cambodia versus Vietnam) (Figure 8), with large differentiation in intercontinental comparisons (Brazil versus Cambodia, *F*_ST _= 0.746; Brazil versus Vietnam, *F*_ST _= 0.755; Brazil versus Sri Lanka, *F*_ST _= 0.528). The low *F*_ST _value estimated for the comparison between Cambodian and Vietnamese parasites is particularly noteworthy, given the long time interval between dates of sample collection in these sites.

We focused on two nonsynonymous substitutions in *pvmdr-1 *thought to be associated with CQ resistance, Y976F and F1076L [21-23]. Double-mutant alleles accounted for all or nearly all samples from Cambodia and Vietnam, while wild-type alleles predominated in Brazil. An intermediate pattern was found in Sri Lanka (Figure 8). All but one single-mutant allele carried the F1076L change; the only allele carrying the Y976F change alone (i. e., not co-occurring with the F1076L change) came from Granada, Brazil. These findings lend further support to the hypothesis that a two-step mutational trajectory (F1076L followed by Y976F) at the *pvmdr-1 *locus leads to CQ resistance [21]. If this hypothesis is correct, molecular detection of F1076L single mutants may provide an early warning about the risk of emerging CQ resistance before the drug-resistant phenotype itself can be detected in populations.

The finding of parasites with wild-type *pvmdr-1 *alleles that are CQ-resistant [22,24] suggests that other polymorphisms may contribute to this phenotype. Similar to *P. falciparum *[25], in vitro susceptibility to CQ in *P. vivax *appears to be modulated by *pvmdr-1 *copy number. Gene amplification correlates with increased susceptibility to CQ and decreased susceptibility to amodiaquine, artesunate and mefloquine [26]. Different country-specific drug policies may therefore favor parasites with increased *pvmdr-1 *copy number or select for Y976F alleles, further complicating geographic comparisons of allele frequencies and associated phenotypes.

### Prospects for genome-wide population association studies

Here we describe long-ranging chromosome-level LD and relatively conserved haplotype blocks in *P. vivax *populations from areas with low to moderate levels of malaria transmission. The most favorable conditions for association studies with relatively low marker density were observed in Brazil, where parasites were reasonably diverse (SNP π values comparable to those estimated for local *P. falciparum *populations) and strong LD was observed at relatively short intermarker distances (~40 kb) but gradually declined with increasing physical distance between pairs of markers. In contrast, the similar levels of LD, along the whole 100-kb region analyzed, in the Asian populations may increase the probability of detecting false-positive associations between phenotype-associated loci and genetic markers located at considerable map distances. These findings, although limited to a single chromosome segment that comprises only 0.4% of the whole genome of the parasite, indicate that genome-wide association studies can represent a feasible strategy to map genetic regions associated with drug resistance, virulence and other phenotypes of interest in carefully selected *P. vivax *populations. Genome-wide studies with higher marker density are required to confirm these findings.

We have also observed substantial genetic differentiation among populations, at both local and regional levels. Consistent with previous studies of *P. falciparum *[27], we found significant geographic structure revealed by synonymous SNPs, which are putatively free of strong directional selection. In addition, we found large differences in the frequencies of nucleotide substitutions at the *pvmdr-1 *locus among populations. We note that, because of the underlying geographic structure, allele frequency differences observed among populations may be unrelated to the genetics of the particular phenotype under study, resulting in false-positive results or reduced power of association studies. Even when studies are restricted to a single continental origin, false positives may still result from major differences in the ancestry of local parasites, such as those found in Brazil. Accounting for the geographic structure is a major practical issue in future population-based studies of genetic determinants of *P. vivax *traits. Statistical methods have now been developed [28] to address this issue when ideally homogeneous (unstructured) parasite populations are not available for sampling.

## Conclusion

We found varying levels of SNP diversity and LD across *P. vivax *populations, with the most favorable conditions for genome-wide association studies, relatively high diversity and strong LD that declines with increasing intermarker distance, observed in Brazil. However, we have also observed substantial genetic differentiation among populations at both local and regional levels, especially in the Brazilian population. Although these results suggest that association studies are feasible in selected *P. vivax *populations, they highlight the need for correcting for population structure to avoid false-positive associations.

## Methods

### Geographical parasite sampling

We collected venous or finger-prick blood samples from 432 patients with slide-confirmed *P. vivax *infection from three locations in Brazil and one location each in three Asian countries (Cambodia, Vietnam, and Sri Lanka (Figure 5). All sites in Brazil are characterized by year-round but hypoendemic malaria transmission, with *P. vivax *prevalence rates typically below 1% [29,30]. The 249 samples from Brazil were collected in the rural settlement of Granada and the town of Plácido de Castro (50 km south of Granada), both in Acre State, and in the city of Porto Velho (500 km east of Granada), in Rondônia State. The samples from Granada (n = 193) were collected during prospective cohort studies between 2004 and 2006 [11,16,30], those from Plácido de Castro (n = 38) were collected in 2008 from patients attending the town's malaria clinic [31], and those from Porto Velho (n = 17) were collected from patients attending the Oswaldo Cruz Outpatient Clinic in June-July 1995 [32]. The 70 samples from Cambodia were collected between June and December 2008 in Pursat town from individuals who became infected in the nearby forests (ClinicalTrials.gov identifier, NCT00663546). In April 2008, a cross-sectional survey of 1056 individuals living in the forest fringe of Pursat province, near the border with Thailand, estimated a *P. vivax *prevalence rate of 2.2% (CA and RMF, unpublished results). The 23 samples from Vietnam were collected in January-December 1995 from patients attending the outpatient clinic of the Lam Dong Provincial Hospital, in the town of Bao Loc, 150 km northwest of Ho Chi Mihn City, Lam Dong Province [33]. Prevalence rates of *P. vivax *infection in the rural communities surrounding Bao Loc, on the southern highlands of Vietnam, were estimated to be around 2.5-7.5% at the time of sample collection [33]. The 29 samples from Sri Lanka were collected during a malaria outbreak in Trincomalee, Eastern Province [34]. Between January and August 2007, a total of 87 cases of *vivax *malaria were reported from Trincomalee (Anti-Malaria Campaign, Ministry of Health, Sri Lanka and TT, unpublished information). Over the past few years malaria transmission has declined steadily in Sri Lanka, from 210,039 cases of malaria in 2001 to only 196 cases country-wide in 2007 [35]. In contrast, no drastic reduction in malaria transmission has been documented over the past decade in the other Asian sites included in this study.

DNA from field samples was extracted using standard protocols referenced in the original publications. Additional DNA samples, from monkey-adapted strains, Belém (isolated in Brazil, 1980) and Salvador-I (El Salvador, 1969), were provided by the Malaria Research and Reference Reagent Resource Center (MR4), ATCC (Manassas, United States). To obtain adequate DNA concentrations for SNP assays, parasite DNA was submitted to whole-genome amplification (WGA) prior to typing. WGA was performed on 10 ng of genomic DNA, with high-fidelity multiple displacement technology [36], using a REPLI-g Minikit (Qiagen, Valencia, United States) according to the manufacturer's instructions.

### Single-nucleotide polymorphisms (SNPs)

We identified SNPs across chromosome 8 by aligning 100 kb of contiguous DNA sequence from five *P. vivax *isolates [37,38] (GenBank accession numbers AY003872 and AY216936-AY216939). DNA sequences were derived from two isolates from Brazil (Belém strain [39] and a field isolate from Rondônia [40]), one from El Salvador (Salvador-I strain [41]), one from India (India VII strain [42]), and one from Thailand (Thai NYU strain [43]). SNPs were selected to fit two criteria: that they are located in nonrepetitive regions and that they are surrounded by 200 bp of upstream sequence and 200 bp of downstream sequence with no significant similarity to human sequences, as determined by BLAST search against the human genome, to prevent cross-amplification of human DNA present in the field-collected test samples. We examined a single chromosome region (spanning ~0.4% of the whole genome) because other genomic regions of *P. vivax *have not been systematically screened for SNPs using a worldwide parasite sample [37]. We designed assays to 108 candidate SNPs meeting the criteria above, which were screened in 50 field-collected samples. Of them, 22 were discarded because alleles were called in less than 10% of these 50 test samples and one was discarded because of cross-amplification from human DNA. The final set comprised 85 markers across chromosome 8, with 75 silent SNPs (39 intergenic, 1 intronic, 9 located in 5' or 3' untranslated regions (UTR) and 26 synonymous nucleotide replacements in open reading frames of genes encoding annotated or hypothetical proteins) and 10 nonsynonymous SNPs (Additional file 8 Table S3).

We also examined nucleotide replacements at two loci encoding digestive-vacuole membrane proteins that are potentially involved in chloroquine (CQ) resistance in *P. vivax *(Additional file 7 Figure S5). Since parasites are exposed to different drug treatment regimes in each country, local adaptation may theoretically result in more geographic structure revealed by these polymorphisms than neutral markers [27]. Although the molecular mechanisms of CQ resistance in *P. vivax *remain unknown, we focused on the two most likely candidate genes. We typed five nonsynonymous SNPs (L47 S, K76T, S250P, F276V, and L384F) in the *pvcrt-o *gene [44] that were recently found in field isolates of *P. vivax *[44,45]. K76T mutant alleles of the *P. falciparum *orthologue of *pvcrt-o*, which encodes the protein chloroquine resistance transporter (PfCRT), confer CQ resistance to this species [46], but the limited data available to date fail to support associations between mutations in *pvcrt-o *alleles and CQ resistance in *P. vivax *[44,45]. We also typed one synonymous (at codon 4065) and five nonsynonymous (N89 S, N500 D, M908L, Y976F, and F1076L) SNPs previously described at the *multidrug resistance 1 *gene of *P. vivax *(*pvmdr-1*) [21,22,24,45], which encodes a P-glycoprotein of the family of ATP binding cassette (ABC) transporters. The Y976F mutation (TAC→TTC) has been associated with CQ resistance in Southeast Asia [22] and Papua New Guinea [23]. Interestingly, the Y976F change is rarely, if ever, observed in alleles that do not carry the F1076L (TTT→CTT) change, suggesting a two-step mutation pathway leading to CQ resistance [21]. All SNPs were genotyped, under contract, by K-Biosciences (Cambridge, UK), with an amplifluor assay [47,48]. Primer sequences for amplifying the SNPs are provided in the additional file 9 Table S4; the annealing temperature for all primers was 60°C. Accuracy of genotyping was empirically assessed as 99.8% in blind replicate analyses of 5493 SNPs.

### Data analysis

Estimation of allele frequencies in malaria parasite populations is complicated by the co-occurrence of multiple clones within infections. Counting all alleles identified within an infection results in overestimation of frequencies of rare alleles and underestimation of common alleles. To minimize bias, we excluded 101 infections in which > 1 allele was observed in any of the SNP loci. For the analysis of chromosome-level SNP diversity and LD patterns, we also excluded 93 infections with allele calls for < 60 markers across chromosome 8. Analysis of the *pvcrt-o *and *pvmdr-1 *loci was based on complete genotypes; infections with one or more SNPs without allele calls were excluded. The number of isolates considered for further analysis is shown in the additional file 1 Table S1.

Population-level diversity was measured with the SNP π statistic, defined as the average number of pairwise differences at assayed SNPs between all members of a population [15]. SNP π values were also calculated separately for silent (synonymous or noncoding) and nonsynonymous SNPs, with standard errors estimated by bootstrapping, and compared with nonparametric Wilcoxon tests. To test whether average SNP π values increased with increased distance between dates of collection of sympatric isolates, we used Poptools (version 2.7.1) software [49] to run a Mantel matrix correlation test [50], with 1000 permutations, on the Granada subpopulation dataset (isolates systematically sampled between 2004 and 2006).

A key factor in the success of association studies is the level of LD observed within and across populations. We examined the evidence for LD within each country and in two subpopulations from Brazil (Granada and Plácido de Castro) with enough samples. The LD statistic *r*^2 ^[51] was calculated for all pairs of SNPs across chromosome 8, within populations, using LDA software [52]. Statistical significance of LD was tested, at the 5% level, using χ^2 ^tests. Correlation between the physical distance between markers and LD was assessed using the Pearson's coefficient of correlation (*r*). We defined chromosome 8 haplotype blocks as clusters of adjacent markers over which evidence of meiotic recombination was minimal, using the four-gamete rule [53]. For each pair of markers, the frequencies of all four combinations of alleles were computed; blocks were built with consecutive markers where only three or less combinations were observed with a frequency ≥ 0.01. Haplotype blocks were generated with Haploview (version 4.1) software [54]. To compare haplotype block boundaries across different populations, we examined the proportions of pairs of markers assigned to the same block (i. e, non-recombining sites) and to different blocks of no block (i. e., recombining sites) in each population. A SNP pair was considered concordant if the assignment was the same in both populations analyzed and discordant if the assignments disagreed [55]. These comparisons were made for pairs of SNPs spaced up to 31 kb, since this is the length of the largest haplotype block found in our study populations.

We assessed population differentiation using the θ estimator [56] of *F*_ST _statistic using FSTAT software [57]; 95% confidence intervals were derived by bootstrapping to determine whether values differed significantly from zero. We used principal component analysis (PCA) to determine whether isolates could be regarded as randomly chosen from a single, genetically homogeneous population or whether they were clustered, defining subpopulations [18]. We carried out separate analysis for the whole dataset and for the populations from Brazil and Asia, using the MeV software [58]. Because strong intermarker LD may distort PCA results [18], we compared clustering patterns obtained with all informative SNPs (e.g., those that segregated in the population under analysis) with the patterns obtained with a filtered SNP set, in which a single marker was selected from every haplotype block detected by Haploview (defined as above). We also employed STRUCTURE 2.2 software [20] to examine parasite population structure. This software uses a Bayesian clustering approach to assign isolates to *K *populations characterized by a set of allele frequencies at each locus. We run the program 10 times each for *K *values between 1 and 6. Each analysis involved 100,000 iterations, with 50,000 burn-in cycles. We used the linkage model to account for the LD among markers across the same chromosome. We computed the posterior probability for each *K *and show here clustering patterns associated with the strongest statistical support. Again, separate analyses were made for the whole dataset and for Brazilian and Asian samples.

### Ethics statement

The parasitized blood samples described in this paper were collected under protocols approved by the relevant ethical review committees in the respective countries. Patients or their parents or legal guardians provided written informed consent before donating samples. Ethical clearance was also obtained from the Institutional Review Board (IRB) of the University of São Paulo, Brazil; the IRB of the NIAID, USA; the National Research Ethics Committee of the Brazilian Ministry of Health; and the Cambodian National Ethics Committee for Health Research.

## Authors' contributions

Conceived and designed the experiments: POS, NDK, DFW, MUF. Performed the experiments: POS. Contributed parasite samples and epidemiologic data: MdSN, NDK, NSdS, KKGS, RMG, CA, JMS, DS, RMF, SG, TT, GG, RA, FK, MUF. Analyzed the data: POS, MUF. Drafted the paper: POS, MUF. All authors read and approved the final version of the manuscript.

## Supplementary Material

Additional file 1 **Table S1**. Origin of field isolates of *P. vivax *analyzed in this study. Click here for file

Additional file 2 **Figure S1**. Variation in LD in relation to distance between SNP loci along chromosome 8 of *P. vivax *in two Brazilian subpopulations. Data are shown for Granada and Plácido de Castro. LD was measured with the *r*^2 ^statistic (values on y-axis are multiplied by 100); closed (blue) circles denote significant LD (χ^2 ^statistic, *P *< 0.05), while open circles denote nonsignificant LD. A significant negative correlation between *r*^2 ^values and physical distance between SNPs was found for both Granada (Pearson correlation coefficient *r *= -0.180, *P *< 0.0001) and Plácido de Castro (*r *= -0.096, *P *= 0.028). Click here for file

Additional file 3 **Table S2**. Matrix of *F*_ST _values showing pairwise comparisons for 10 nonsynonymous SNPs (above diagonal) and 75 silent SNPs (below diagonal). Click here for file

Additional file 4 **Figure S2**. Principal component analysis (PCA) of population structure of *P. vivax*. This analysis considered a subset of 29 SNPs selected to minimize intermarker LD. Plots show the three first principal components. Each circle or square represents a parasite isolate or strain and the color is assigned according to the geographic origin of parasites: green circles, Granada; red circles, Plácido de Castro; open circles, Porto Velho (all samples from Brazil are represented with circles); pink squares, Cambodia; blue squares, Sri Lanka; light brown squares, Vietnam (all samples from Asia are represented with squares). The strains Salvador I and Belém are denoted by orange and black circles, respectively. Upper panel, plot of the first two principal components; lower panel, plot of the first and third principal components. Note that the clustering patterns revealed by the subset of 29 SNPs are quite similar to that detected with 57 informative SNPs (Figure 6). Click here for file

Additional file 5 **Figure S3**. Principal component analysis (PCA) of population structure of *P. vivax *in Brazil. Plots show the three first principal components. Each circle represents a parasite isolate or strain and the color is assigned according to the geographic origin of parasites: green, Granada; red, Plácido de Castro; open, Porto Velho. The strains Salvador I and Belém are denoted by orange and black colors, respectively. Upper panel, plot of the first two principal components; lower panel, plot of the first and third principal components. Click here for file

Additional file 6 **Figure S4**. Principal component analysis (PCA) of population structure of *P. vivax *in Asia. Plots show the three first principal components. Each square represents a parasite isolate and the color is assigned according to the geographic origin of parasites: pink, Cambodia; blue, Sri Lanka; light brown, Vietnam. Upper panel, plot of the first two principal components; lower panel, plot of the first and third principal components. Click here for file

Additional file 7 **Figure S5**. Predicted structure and location of amino acid replacements in PvMDR-1 and PvCRT-O. PvMDR-1 (upper panel) has two hydrophobic domains, each with six transmembrane α-helices, and a cytosolic domain harboring nucleotide-binding domains 1 (NBD_1_) and NBD_2_, each containing an ATP-binding site. PvCRT-O (lower panel) has 10 predicted transmembrane helices, with C- and N-terminal domains located in the parasite cytoplasm. Black dots represent nonsynonymous SNPs assayed in this study; the only open dot in PvMDR-1 represents a synonymous SNP that was also assayed. Click here for file

Additional file 8 **Table S3**. Characterization of the 85 biallelic single-nucleotide polymorphisms (SNPs) along *P. vivax *chromosome 8 assayed in this study. Click here for file

Additional file 9 **Table S4**. Sequences of primers used to amplify 85 single-nucleotide polymorphisms (SNPs) along chromosome 8 of *P. vivax*, 5 SNPs in the *pvcrt-o *gene and 6 SNPs in the *pvmdr-1 *gene. Click here for file
