# Supplementary material for: Single-nucleotide polymorphism, linkage disequilibrium and geographic structure in the malaria parasite Plasmodium vivax: prospects for genome-wide association studies
Source: BMC Genet. 2010 Jul 13;11:65. doi: 10.1186/1471-2156-11-65 (PMC2910014; doi:10.1186/1471-2156-11-65)
Supplement: Additional file 1 — Table S1. Origin of field isolates of P. vivax analyzed in this study. [file 1471-2156-11-65-S1.DOC]

**Additional file 1 Table S1.** Origin of field isolates of *P. vivax* analyzed in this study.

|  |  |  | **No. of field isolates assayed for SNPs** | | |
| --- | --- | --- | --- | --- | --- |
| **Country** | **Malaria endemicity** | **Site and years of sample collection** | **Chromosome 8** | ***pvmdr-1*** | ***pvcrt-o*** |
| Brazil | Low | Granada, 2004-06 | 119 | 111 | 102 |
|  |  | Plácido de Castro, 2008 | 35 | 37 | 34 |
|  |  | Porto Velho, 1995 | 8 | 8 | 8 |
| Cambodia | Moderate | Pursat, 2008 | 41 | 69 | 62 |
| Sri Lanka | Very low | Trincomalee, 2007 | 21 | 20 | 19 |
| Vietnam | Moderate | Bao Loc, 1995 | 14 | 9 | 7 |
