# Supplementary figures and images for: Single-nucleotide polymorphism, linkage disequilibrium and geographic structure in the malaria parasite Plasmodium vivax: prospects for genome-wide association studies
Source: BMC Genet. 2010 Jul 13;11:65. doi: 10.1186/1471-2156-11-65 (PMC2910014; doi:10.1186/1471-2156-11-65)

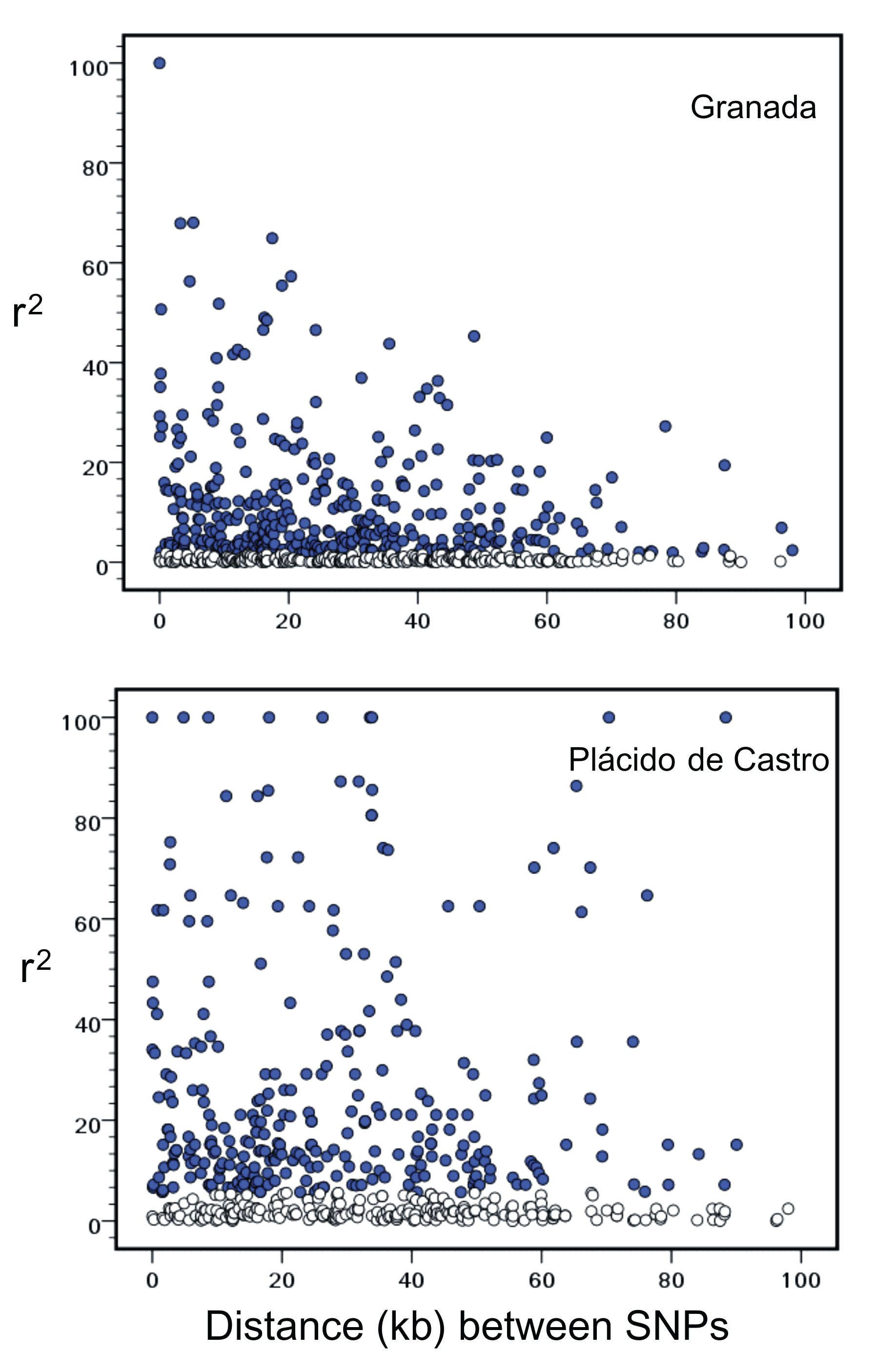

Supplement: Additional file 2 — Figure S1. Variation in LD in relation to distance between SNP loci along chromosome 8 of P. vivax in two Brazilian subpopulations. Data are shown for Granada and Plácido de Castro. LD was measured with the r2 statistic (values on y-axis are multiplied by 100); closed (blue) circles denote significant LD (χ2 statistic, P < 0.05), while open circles denote nonsignificant LD. A significant negative correlation between r2 values and physical distance between SNPs was found for both Granada (Pearson correlation coefficient r = -0.180, P < 0.0001) and Plácido de Castro (r = -0.096, P = 0.028). [file 1471-2156-11-65-S2.TIFF]

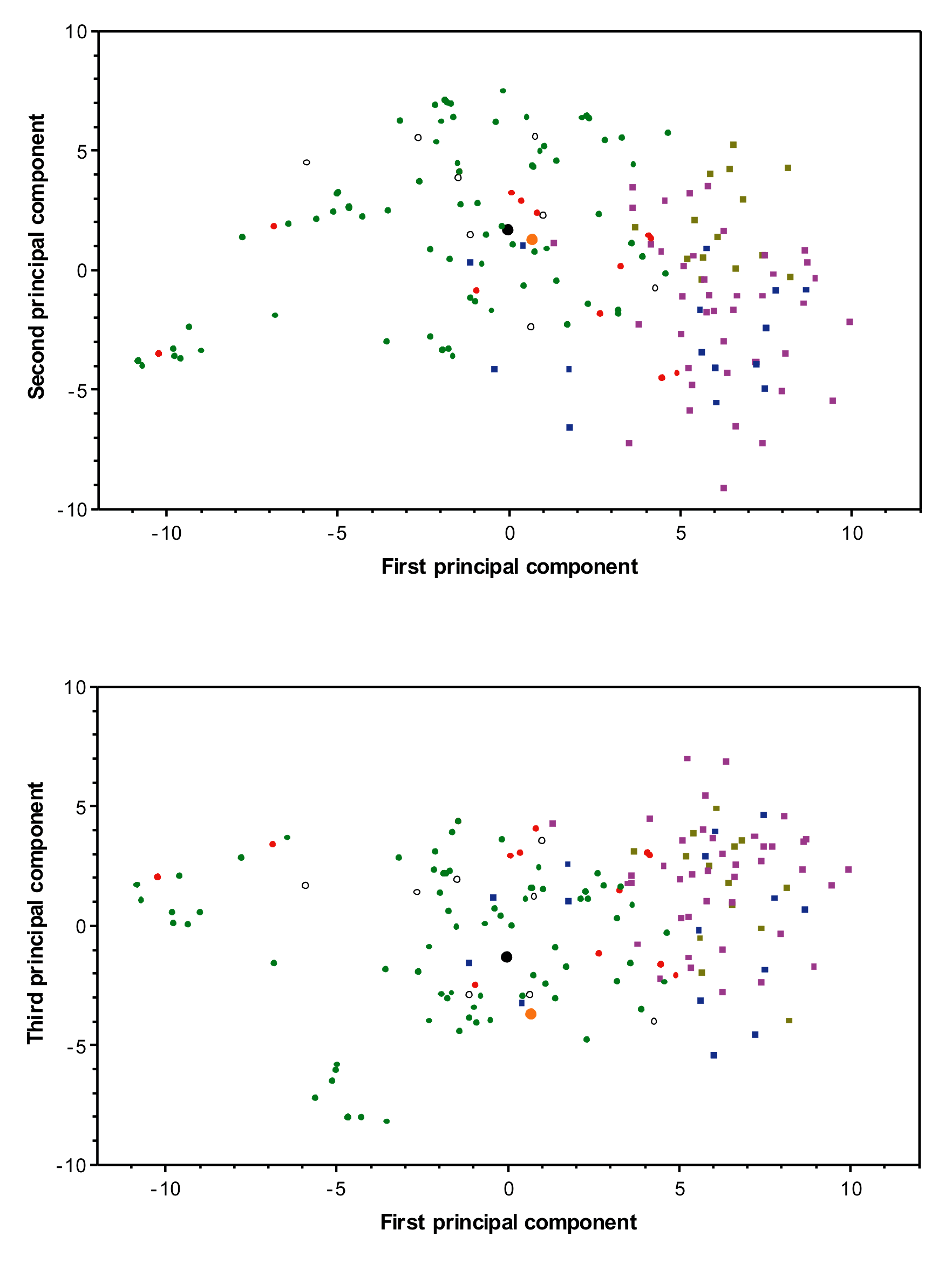

Supplement: Additional file 4 — Figure S2. Principal component analysis (PCA) of population structure of P. vivax. This analysis considered a subset of 29 SNPs selected to minimize intermarker LD. Plots show the three first principal components. Each circle or square represents a parasite isolate or strain and the color is assigned according to the geographic origin of parasites: green circles, Granada; red circles, Plácido de Castro; open circles, Porto Velho (all samples from Brazil are represented with circles); pink squares, Cambodia; blue squares, Sri Lanka; light brown squares, Vietnam (all samples from Asia are represented with squares). The strains Salvador I and Belém are denoted by orange and black circles, respectively. Upper panel, plot of the first two principal components; lower panel, plot of the first and third principal components. Note that the clustering patterns revealed by the subset of 29 SNPs are quite similar to that detected with 57 informative SNPs (Figure 6). [file 1471-2156-11-65-S4.TIFF]

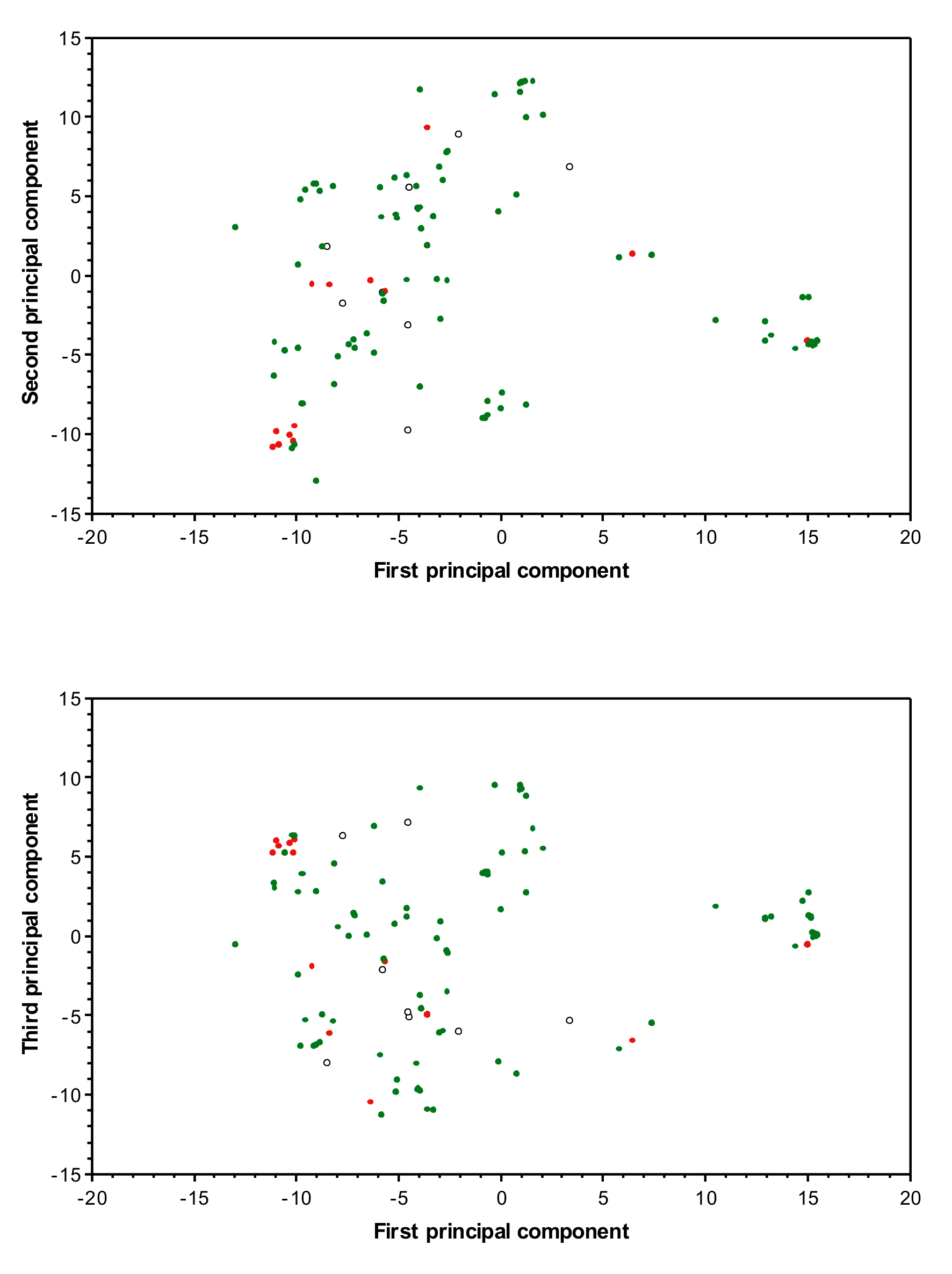

Supplement: Additional file 5 — Figure S3. Principal component analysis (PCA) of population structure of P. vivax in Brazil. Plots show the three first principal components. Each circle represents a parasite isolate or strain and the color is assigned according to the geographic origin of parasites: green, Granada; red, Plácido de Castro; open, Porto Velho. The strains Salvador I and Belém are denoted by orange and black colors, respectively. Upper panel, plot of the first two principal components; lower panel, plot of the first and third principal components. [file 1471-2156-11-65-S5.TIFF]

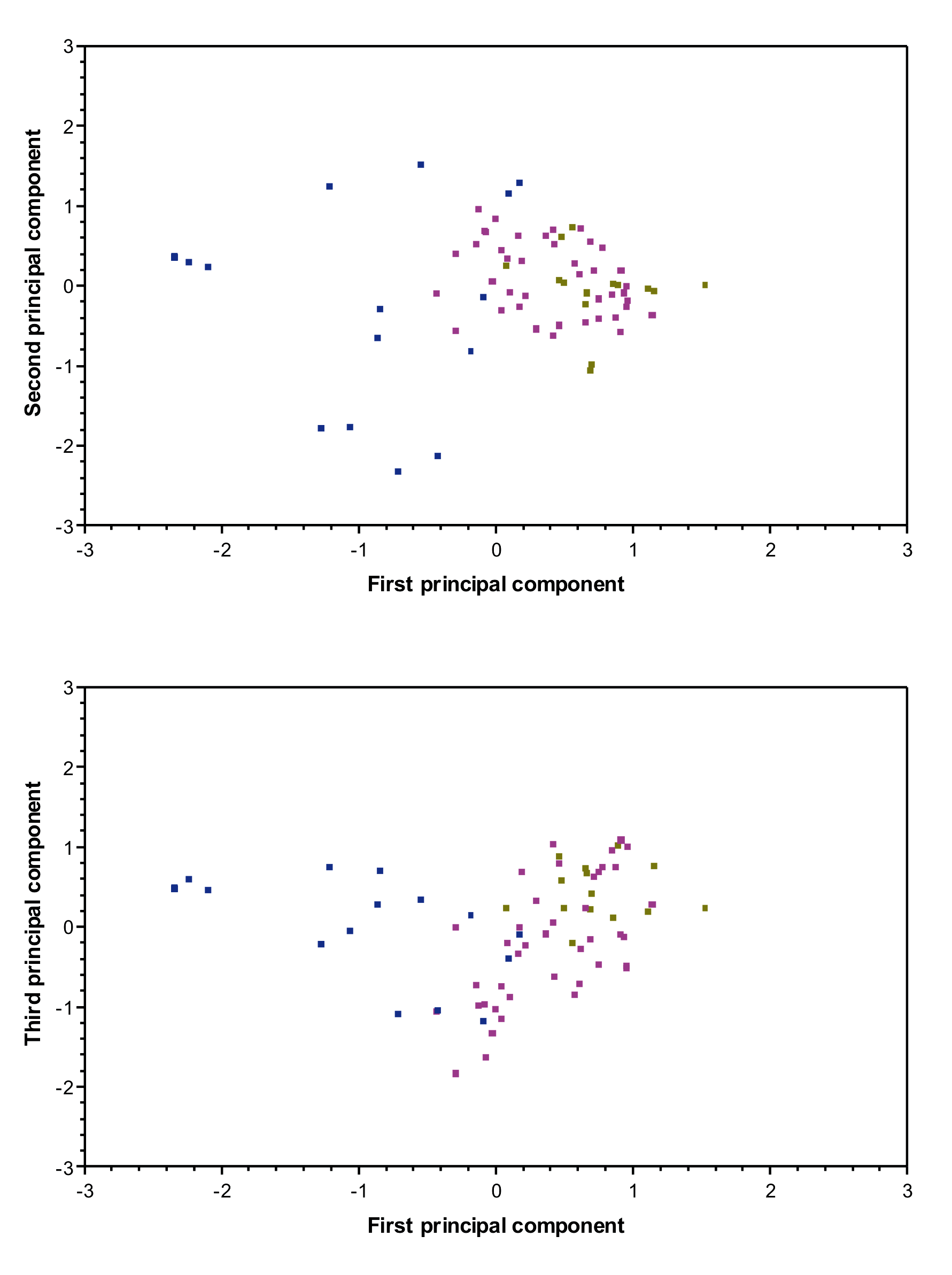

Supplement: Additional file 6 — Figure S4. Principal component analysis (PCA) of population structure of P. vivax in Asia. Plots show the three first principal components. Each square represents a parasite isolate and the color is assigned according to the geographic origin of parasites: pink, Cambodia; blue, Sri Lanka; light brown, Vietnam. Upper panel, plot of the first two principal components; lower panel, plot of the first and third principal components. [file 1471-2156-11-65-S6.TIFF]

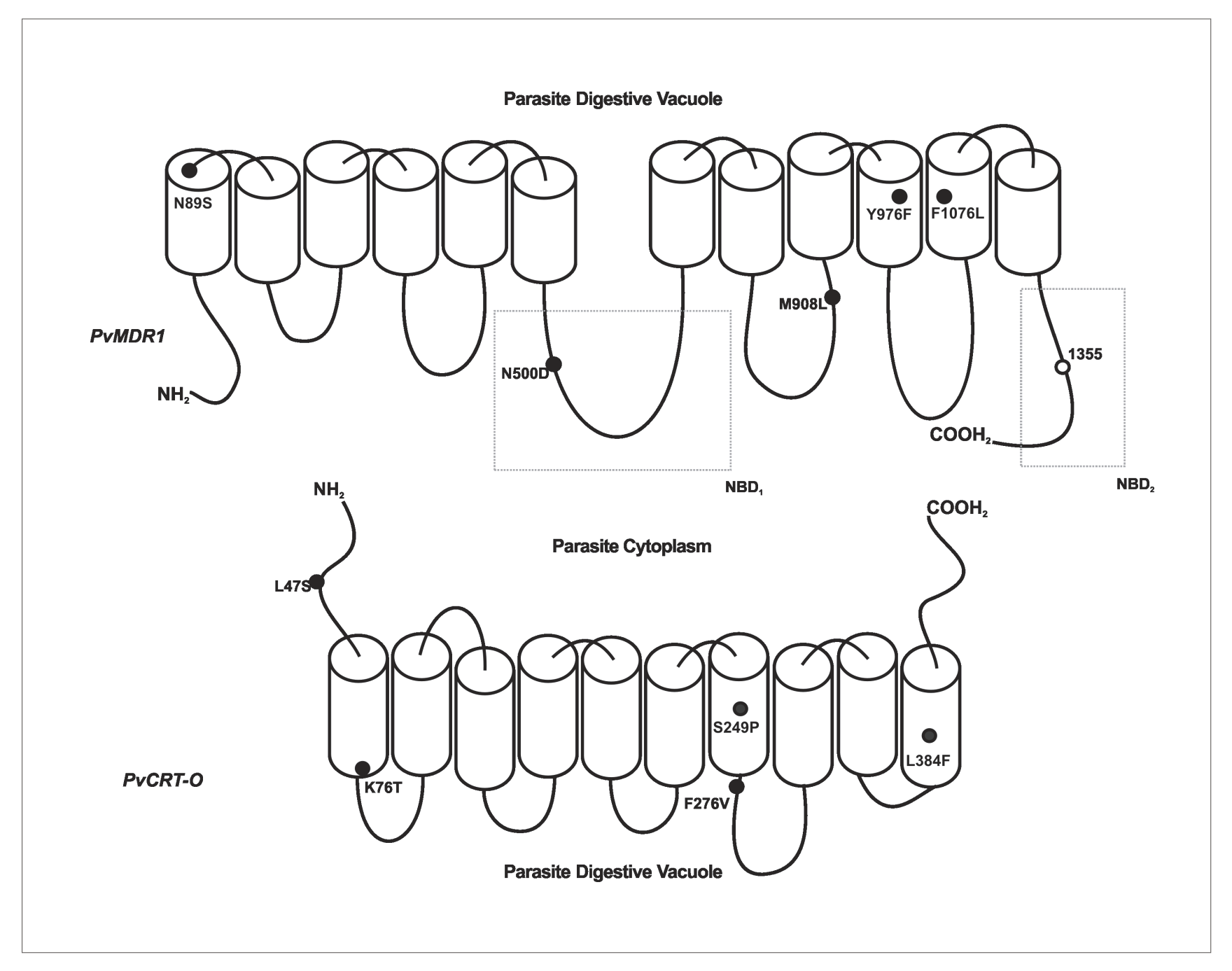

Supplement: Additional file 7 — Figure S5. Predicted structure and location of amino acid replacements in PvMDR-1 and PvCRT-O. PvMDR-1 (upper panel) has two hydrophobic domains, each with six transmembrane α-helices, and a cytosolic domain harboring nucleotide-binding domains 1 (NBD1) and NBD2, each containing an ATP-binding site. PvCRT-O (lower panel) has 10 predicted transmembrane helices, with C- and N-terminal domains located in the parasite cytoplasm. Black dots represent nonsynonymous SNPs assayed in this study; the only open dot in PvMDR-1 represents a synonymous SNP that was also assayed. [file 1471-2156-11-65-S7.TIFF]
