# Supplementary material for: Single-nucleotide polymorphism, linkage disequilibrium and geographic structure in the malaria parasite Plasmodium vivax: prospects for genome-wide association studies
Source: BMC Genet. 2010 Jul 13;11:65. doi: 10.1186/1471-2156-11-65 (PMC2910014; doi:10.1186/1471-2156-11-65)
Supplement: Additional file 3 — Table S2. Matrix of FST values showing pairwise comparisons for 10 nonsynonymous SNPs (above diagonal) and 75 silent SNPs (below diagonal). [file 1471-2156-11-65-S3.DOC]

**Additional file 3 Table S2.**  Matrix of *F*_ST_ values showing pairwise comparisons for 10 nonsynonymous SNPs (above diagonal) and 75 silent SNPs (below diagonal). Asterisks indicate values significantly different from zero (*P* < 0.05).

| Country | Brazil | Cambodia | Sri Lanka | Vietnam |
| --- | --- | --- | --- | --- |
| Brazil | - | 0.225* | 0.360* | 0.188* |
| Cambodia | 0.421* | - | 0.293* | 0.031 |
| Sri Lanka | 0.427* | 0.221* | - | 0.276* |
| Vietnam | 0.405* | 0.092* | 0.299* | - |
