# Supplementary material for: Single-nucleotide polymorphism, linkage disequilibrium and geographic structure in the malaria parasite Plasmodium vivax: prospects for genome-wide association studies
Source: BMC Genet. 2010 Jul 13;11:65. doi: 10.1186/1471-2156-11-65 (PMC2910014; doi:10.1186/1471-2156-11-65)
Supplement: Additional file 8 — Table S3. Characterization of the 85 biallelic single-nucleotide polymorphisms (SNPs) along P. vivax chromosome 8 assayed in this study. [file 1471-2156-11-65-S8.DOC]

**Additional file 8 Table S3.**  85 biallelic single-nucleotide polymorphisms (SNPs) along *P. vivax* chromosome 8 assayed in this study.

| **SNP** | **Chromosome location**  **(GenBank NC_009913.1)** | **Locus** | **Location*** | **Type of SNP** |
| --- | --- | --- | --- | --- |
| C/T | 1623958 | Intergenic | Intergenic | Silent |
| A/G | 1622285 | Intergenic | Intergenic | Silent |
| C/T | 1622111 | Intergenic | Intergenic | Silent |
| C/T | 1619728 | ATP-dependent transporter | ORF | Silent |
| A/G | 1618374 | ATP-dependent transporter | ORF | Nsyn |
| C/G | 1614542 | Hypothetical protein | 5' UTR | Silent |
| A/G | 1614205 | Intergenic | Intergenic | Silent |
| A/C | 1613464 | Intergenic | Intergenic | Silent |
| A/G | 1613391 | Intergenic | Intergenic | Silent |
| A/G | 1610174 | Intergenic | Intergenic | Silent |
| G/T | 1609953 | Intergenic | Intergenic | Silent |
| A/G | 1604284 | Exportin 1 | ORF | Silent |
| A/T | 1602879 | Intergenic | Intergenic | Silent |
| A/G | 1600724 | N-Ethylmaleimide-sensitive fusion protein NSF | ORF | Silent |
| A/C | 1600723 | N-Ethylmaleimide-sensitive fusion protein NSF | ORF | Silent |
| A/G | 1598306 | N-Ethylmaleimide-sensitive fusion protein NSF | ORF | Silent |
| C/T | 1597711 | Intergenic | Intergenic | Silent |
| C/T | 1597701 | Intergenic | Intergenic | Silent |
| C/T | 1597561 | Intergenic | Intergenic | Silent |
| C/G | 1597560 | Intergenic | Intergenic | Silent |
| C/G | 1597489 | Intergenic | Intergenic | Silent |
| C/G | 1596010 | Hypothetical protein | ORF | Silent |
| C/G | 1593850 | Hypothetical protein | ORF | Silent |
| G/T | 1592890 | Hypothetical protein | 5' UTR | Silent |
| A/C | 1592740 | Hypothetical protein | 5' UTR | Silent |
| C/T | 1584593 | DNA-directed RNA polymerases I, II, and III 14.4 | 3' UTR | Silent |
| C/T | 1584352 | DNA-directed RNA polymerases I, II, and III 14.4 | ORF | Silent |
| C/G | 1582289 | Intergenic | Intergenic | Silent |
| C/T | 1582190 | Intergenic | Intergenic | Silent |
| C/T | 1581568 | Intergenic | Intergenic | Silent |
| C/T | 1581478 | Intergenic | Intergenic | Silent |
| G/T | 1578753 | Intergenic | Intergenic | Silent |
| A/G | 1578363 | Intergenic | Intergenic | Silent |
| A/G | 1576155 | Hypothetical protein | ORF | Silent |
| C/T | 1576132 | Hypothetical protein | ORF | Silent |
| A/G | 1575875 | Hypothetical protein | ORF | Nsyn |
| A/G | 1573532 | Hypothetical protein | ORF | Silent |
| C/G | 1572490 | Hypothetical protein | ORF | Silent |
| A/G | 1569656 | Intergenic | Intergenic | Silent |
| C/T | 1567898 | Intergenic | Intergenic | Silent |
| A/G | 1567585 | Intergenic | Intergenic | Silent |
| A/T | 1565910 | Hypothetical protein | ORF | Silent |
| A/G | 1565508 | Hypothetical protein | ORF | Nsyn |
| A/G | 1564763 | Intergenic | Intergenic | Silent |
| A/G | 1564543 | Hypothetical protein | 3' UTR | Silent |
| A/G | 1563770 | Intergenic | Intergenic | Silent |
| G/T | 1562732 | Intergenic | Intergenic | Silent |
| G/T | 1562163 | Membrane skeletal protein | ORF | Nsyn |
| C/T | 1562135 | Membrane skeletal protein | ORF | Nsyn |
| G/T | 1561354 | Membrane skeletal protein | ORF | Nsyn |
| C/T | 1561211 | Membrane skeletal protein | ORF | Silent |
| A/G | 1559342 | Membrane skeletal protein | ORF | Silent |
| GC | 1557466 | Intergenic | Intergenic | Silent |
| A/C | 1555684 | Intergenic | Intergenic | Silent |
| A/C | 1554627 | Hypothetical protein | ORF | Silent |
| C/T | 1554609 | Hypothetical protein | ORF | Silent |
| A/T | 1554289 | Hypothetical protein | ORF | Silent |
| A/T | 1553437 | Hypothetical protein | 3' UTR | Silent |
| C/G | 1553420 | Hypothetical protein | 3' UTR | Silent |
| C/T | 1548041 | Intergenic | Intergenic | Silent |
| A/G | 1547536 | Intergenic | Intergenic | Silent |
| C/G | 1547530 | Intergenic | Intergenic | Silent |
| A/G | 1544409 | Hypothetical protein | ORF | Nsyn |
| A/C | 1541670 | 60S ribosomal protein L44 | 5' UTR | Silent |
| C/G | 1541310 | 1-cys-glutaredoxin-like protein-1 | 3' UTR | Silent |
| A/T | 1539632 | Intergenic | Intergenic | Silent |
| C/T | 1538706 | Circumsporozoite (CS) protein | ORF | Silent |
| A/G | 1536248 | Intergenic | Intergenic | Silent |
| A/C | 1536031 | Intergenic | Intergenic | Silent |
| A/C | 1535830 | Intergenic | Intergenic | Silent |
| C/T | 1535808 | Intergenic | Intergenic | Silent |
| G/T | 1533921 | Hypothetical protein | ORF | Nsyn |
| C/T | 1533434 | Hypothetical protein | ORF | Silent |
| A/C | 1533345 | Hypothetical protein | ORF | Nsyn |
| A/C | 1533342 | Hypothetical protein | ORF | Nsyn |
| C/T | 1532019 | Intergenic | Intergenic | Silent |
| A/G | 1531528 | Intergenic | Intergenic | Silent |
| A/G | 1531432 | Intergenic | Intergenic | Silent |
| A/G | 1530686 | Hypothetical protein | ORF | Silent |
| A/C | 1530451 | Hypothetical protein | ORF | Silent |
| A/G | 1529938 | Hypothetical protein | ORF | Silent |
| A/G | 1529698 | Hypothetical protein | ORF | Silent |
| GA | 1527531 | Hypothetical protein | ORF | Silent |
| G/T | 1525970 | Elongation factor TS (EF-TS) | ORF | Silent |
| A/T | 1525816 | Elongation factor TS (EF-TS) | Intron | Silent |

* ORF = open reading frame, 5' UTR = five prime untranslated region, 3' UTR = three prime untranslated region, Nsyn = nonsynonymous substitution.
